# Atypical presentation of systemic lupus erythematosus flare with ileocecal ulceration and lymphadenopathy in a tuberculosis-endemic region

**DOI:** 10.1097/MD.0000000000044964

**Published:** 2025-10-17

**Authors:** Tien Manh Huynh, Nguyen Khoi Huynh, Tran Luong Thi Vo, Huong Tu Lam, Thinh Duc Nguyen, Dong-Hoon Yang

**Affiliations:** a Department of Internal Medicine, School of Medicine, University of Medicine and Pharmacy at Ho Chi Minh City, Ho Chi Minh City, Vietnam; b Department of Gastroenterology, Cho Ray Hospital, Ho Chi Minh City, Vietnam; c Department of Geriatrics and Gerontology, School of Medicine, University of Medicine and Pharmacy at Ho Chi Minh City, Ho Chi Minh City, Vietnam; d Department of Rheumatology, University Medical Center, Ho Chi Minh City, Vietnam; e Department of Endoscopy, Cho Ray Hospital, Ho Chi Minh City, Vietnam; f Department of Internal Medicine, University of Ulsan College of Medicine, Seoul, Korea.

**Keywords:** differential diagnosis, enteritis, lymphadenopathy, systemic lupus erythematosus flare, tuberculosis

## Abstract

**Rationale:**

Systemic lupus erythematosus can involve the gastrointestinal tract, but lupus enteritis (LE) with ileocecal ulceration and generalized lymphadenopathy as an initial presentation is exceptionally rare. In regions where tuberculosis (TB) is endemic, overlapping clinical and imaging findings can lead to diagnostic delays and inappropriate treatment. This case highlights the challenges in differentiating lupus enteritis from abdominal TB and underscores the importance of considering autoimmune etiologies in atypical gastrointestinal presentations.

**Patient concerns:**

A 20-year-old woman presented with acute right lower quadrant abdominal pain, solitary ulceration of the ileocecal valve observed during colonoscopy, and generalized lymphadenopathy involving the abdomen, thorax, and inguinal areas.

**Diagnoses:**

The diagnosis of lupus enteritis was initially complicated and delayed. Nonspecific endoscopic findings, widespread lymphadenopathy, and an indeterminate result from the tuberculosis interferon-gamma release assay (TB-IGRA) raised suspicion for tuberculosis or lymphoma. Further obscuring the diagnosis, a false-negative antinuclear antibody (ANA) test was obtained using the enzyme-linked immunosorbent assay technique. However, persistent gastrointestinal symptoms, markedly elevated levels of anti-double-stranded DNA antibodies, decreased complement levels, and negative tuberculosis testing ultimately supported the diagnosis of lupus enteritis. Confirmation was achieved through an antinuclear antibody immunoblot test.

**Interventions:**

The patient received intravenous methylprednisolone at a dose of 80 milligrams per day, followed by tapering of corticosteroids and maintenance therapy with hydroxychloroquine and azathioprine.

**Outcomes:**

Clinical symptoms resolved within 48 hours. Inflammatory markers normalized within 1 week. At 1-year follow-up, the patient remained in remission, and follow-up colonoscopy confirmed mucosal healing.

**Lessons:**

This case highlights that in TB-endemic regions, clinicians should consider lupus enteritis as a differential diagnosis in patients with nonspecific gastrointestinal lesions and lymphadenopathy, even when tuberculosis is a common concern. False-negative ANA ELISA and indeterminate TB-IGRA results can delay diagnosis. Lymphadenopathy, although not included in current lupus classification criteria, may be an important clinical clue for lupus flares.

## 1. Introduction

Lupus enteritis (LE), a rare complication of systemic lupus erythematosus (SLE), affects to 1% of SLE patients with abdominal pain, contributing to up to 65% of acute abdomen cases in this group.^[1]^ Gastrointestinal involvement occurs in 8% to 40% of SLE patients, with LE in up to 53%, predominantly affecting the ileum (85%).^[2,3]^ In Asia, where SLE prevalence is higher (30–100 per 100,000), LE is increasingly reported, particularly in China and India.^[3,4]^ In contrast, intestinal tuberculosis (ITB) presents with chronic abdominal pain, fever, and weight loss, often involving the ileocecal region.^[5]^ In tuberculosis-endemic Vietnam, distinguishing these conditions is critical yet challenging due to clinical overlap. LE as the initial SLE manifestation is rare, especially in Vietnam, where misdiagnosis as ITB can delay treatment. We report a 20-year-old female with ileocecal ulceration and lymphadenopathy as the sole initial signs of LE, highlighting diagnostic and therapeutic challenges.

## 2. Case presentation

A 20-year-old unmarried female presented with a 3-week history of dull, persistent right lower quadrant abdominal pain accompanied by nonbloody diarrhea occurring 8 to 10 times daily, vomiting 2 to 3 episodes per day, low-grade fever, dry cough, and an unintentional weight loss of 5 kg (from 45 to 40 kg). She denied any dermatological manifestations, alopecia, oral ulcers, hematuria, pleuritic chest pain, arthralgia, or family history of autoimmune diseases. There was no known recent exposure to tuberculosis.

Initial evaluation at a primary care facility revealed a normal chest radiograph except for basal interstitial changes, a negative antinuclear antibody (ANA) by ELISA, and an indeterminate interferon-gamma release assay. Empiric antibiotic therapy (ciprofloxacin and metronidazole) in 7 days yielded no clinical improvement. Colonoscopy demonstrated ulcerations in the terminal ileum, while abdominal ultrasonography identified multiple enlarged lymph nodes, raising suspicion for gastrointestinal tuberculosis and tuberculous lymphadenitis, leading to referral for further evaluation.

On admission, vital signs were within normal limits: temperature 36.7°C, heart rate 92 beats per minute, blood pressure 110/70 mm Hg, respiratory rate 18 breaths per minute, and oxygen saturation 100% on room air. Physical examination revealed mild abdominal distension with normal bowel sounds and localized tenderness in the right lower quadrant without guarding or rebound tenderness.

Laboratory investigations showed a normal leukocyte count (5.6 × 10^3^/µL), lymphopenia (500/µL), normocytic anemia (hemoglobin 8.5 g/dL), thrombocytopenia (101 × 10^3^/µL), hypoalbuminemia (3.1 g/dL), iron deficiency reflected by low serum iron (4.0 µmol/L), reduced transferrin saturation (10.54%), and decreased transferrin levels (151.0 mg/dL). Liver and renal function tests were within normal limits. Bone marrow aspiration revealed findings consistent with peripheral platelet destruction and iron deficiency anemia.

**Figure 1.**
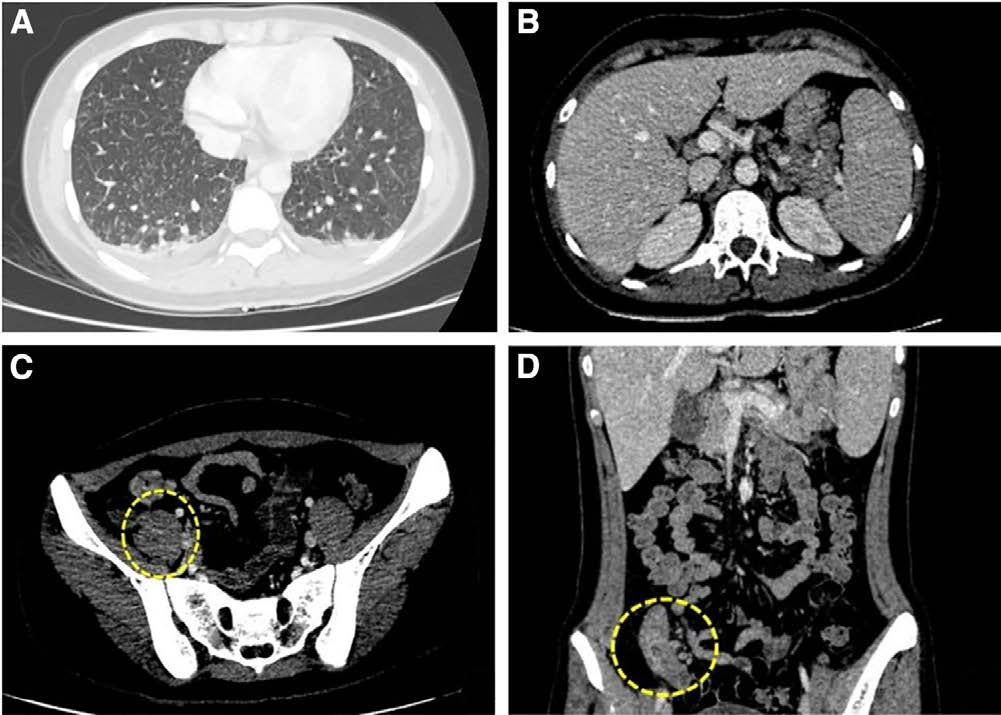
Imaging findings in a 20-year-old female with LE. (A) Chest CT showing reticular patterns and subpleural interlobular septal thickening, indicative of lupus-associated interstitial lung changes similar to UIP, without cavitary lesions. (B–D) Contrast-enhanced abdominal CT demonstrating diffuse non-necrotic lymphadenopathy (red arrows, B), inflammation of the terminal ileum with circumferential and symmetrical mural thickening of the terminal ileum with mild surrounding fat stranding (yellow circles, C), and partial narrowing at the ileocecal valve (yellow dashed circle, D). CT = computed tomography, LE = lupus enteritis, UIP = usual interstitial pneumonia.

**Figure 2.**
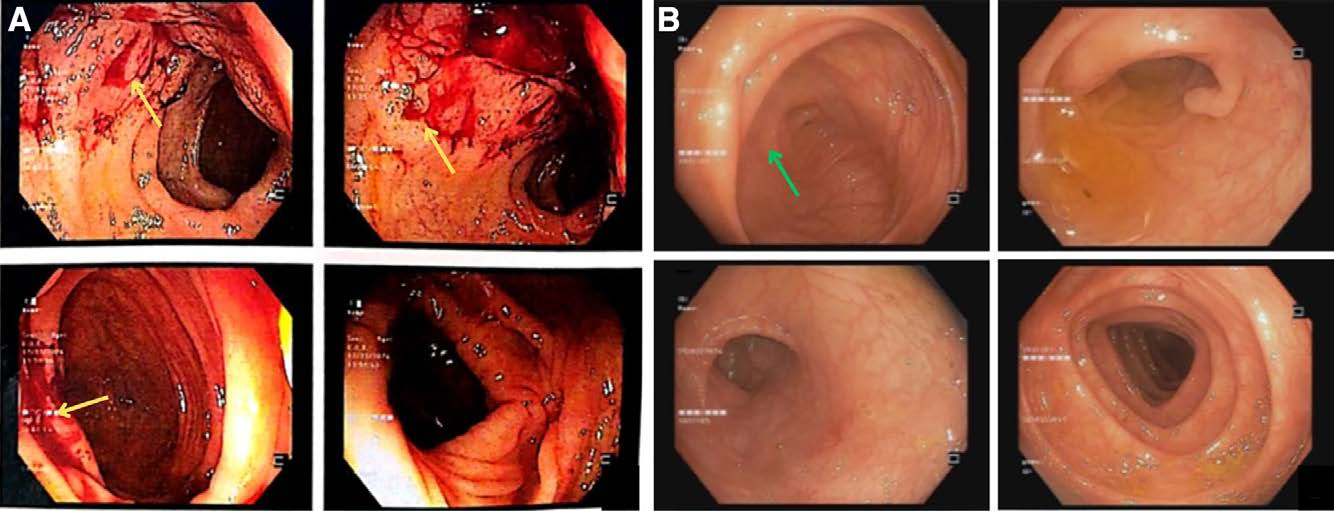
Colonoscopy findings in a 20-year-old female with lupus enteritis. (A) At admission, erosive, ulcerations at the ileocecal valve and superficial ulcers in the terminal ileum (yellow arrows), suggestive of lupus enteritis. (B) At 3-month follow-up, complete mucosal healing with smooth, intact mucosa (green arrows), indicating an excellent response to immunosuppressive therapy.

Chest CT showed reticular and subpleural septal thickening without cavitation, consistent with interstitial lung involvement (Fig. 1A). Abdominal contrast-enhanced CT revealed diffuse non-necrotic lymphadenopathy in the perihepatic and perisplenic regions (Fig. 1B), nonspecific mural thickening of the terminal ileum (Fig. 1C), and partial narrowing at the ileocecal valve (Fig. 1D). Gastroscopy findings were unremarkable. Colonoscopy confirmed ulcerations at the ileocecal valve and terminal ileum (Fig. 2A).

Comprehensive infectious workup was positive solely for *Helicobacter pylori* antigen. Tests for malaria, dengue virus, hepatitis B and C viruses, HIV, routine stool microscopy, bacteriology (including *Clostridioides difficile, Salmonella, Campylobacter, Yersinia* species, parasites), virology, acid-fast bacilli staining, Mycobacterium tuberculosis PCR from sputum, gastric aspirate, and bronchoalveolar lavage fluid were all negative. Urinalysis revealed mild proteinuria (1.1 g/24 hours). Initial autoimmune workup revealed a negative ANA by ELISA despite markedly elevated anti-double-stranded DNA (anti-dsDNA) titers (>240 IU/mL), hypocomplementemia (C3 66 mg/dL, C4 15 mg/dL), and markedly raised inflammatory markers (ESR 130 mm/h, CRP 117 mg/L). Given the constellation of clinical and laboratory findings, and despite the discordant ANA result, a systemic autoimmune process – particularly SLE was strongly suspected. Consequently, comprehensive immunological profiling was pursued. Within 72 hours of presentation to our center, ANA immunoblot confirmed high-titer positivity for anti-dsDNA, nucleosome, histone, and Ku antibodies, consistent with a diagnosis of SLE. Notably, antibodies against SS-A, SS-B, Ro-52, and RNP/Sm were negative.

**Figure 3.**
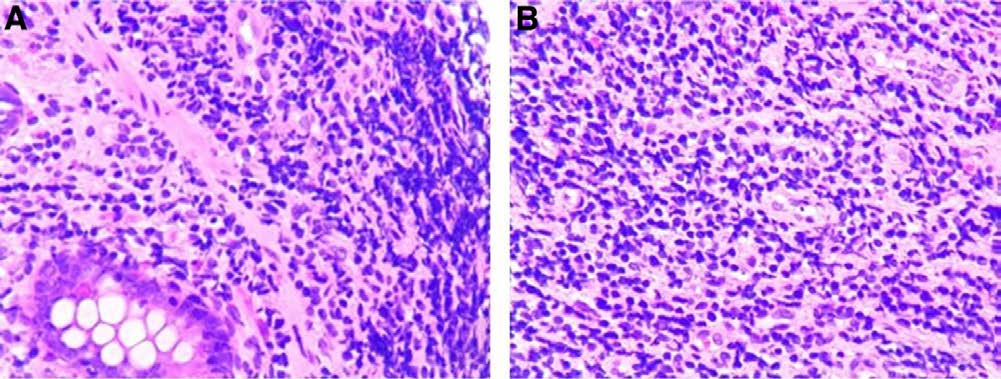
Histopathology of ileocecal biopsy. (A) H&E staining at ×10 magnification showing benign glands with chronic inflammatory infiltrates. (B) H&E at ×40 magnification confirming absence of granulomas or caseous necrosis.

After 7 days, histopathological examination revealed nonspecific chronic inflammation without granulomas or caseous necrosis (Fig. 3A and B), supporting a nontuberculous etiology. The Systemic Lupus Erythematosus Disease Activity Index (SLEDAI) was calculated at 21, indicating very high disease activity, with contributions from vasculitis (8 points), proteinuria (4 points), low complement (2 points), anti-dsDNA (2 points), fever (1 point), and thrombocytopenia (4 points).

The patient responded rapidly to intravenous methylprednisolone (80 mg/d), with abdominal pain, diarrhea, and fever resolving within 48 hours. By day 5, laboratory parameters showed marked improvement: anti-dsDNA decreased to 65 IU/mL, C3 increased from 66 to 99 mg/dL, and C4 to 24 mg/dL. ESR decreased to 56 mm/h, and CRP normalized to 0.7 mg/L. Platelet count recovered to 186 × 10^3^/μL. Follow-up ultrasound showed complete resolution of abdominal lymphadenopathy. She was discharged on a tapering dose of corticosteroids with hydroxychloroquine and azathioprine, and remained in clinical remission at 1-year follow-up, with complete mucosal healing confirmed by colonoscopy at 3 months (Fig. 2B).

## 3. Discussion

LE is defined as vasculitis or inflammation of the small bowel, confirmed by supportive imaging or biopsy findings.^[3,4]^ This condition predominantly involves the jejunum (76%–83%) and ileum (73%–84%) and carries risks of severe complications such as perforation, hemorrhage, or infarction.^[6]^ Our case presents a 20-year-old female with ileocecal ulceration and lymphadenopathy as the sole initial manifestation of an SLE flare – a presentation reported in 13% to 48.5% of LE cases.^[2]^ The diagnosis was delayed for up to 3 weeks at the primary healthcare level due to an indeterminate TB-IGRA result, a false-negative ANA, and a failure to recognize lymphadenopathy as a sign of a lupus flare.

In tuberculosis-endemic, ITB closely mimics LE, particularly since 90% of ITB cases involve the ileocecal region due to abundant lymphoid tissue and physiologic stasis.^[7,8]^ Both conditions may present with abdominal pain, low-grade fever, and lymphadenopathy. However, TB typically features night sweats and weight loss, which were absent in our patient. Misdiagnosis is common as patients with low-grade fever and widespread lymphadenopathy are often treated as abdominal TB with lymph node involvement. TB lymph nodes typically exhibit necrosis, whereas lupus-associated lymphadenopathy is inflammatory, diffuse, and treatment-responsive. Generalized lymphadenopathy may indicate a disease flare but is often missed due to its absence from the EULAR criteria, complicating timely and appropriate management by non-rheumatologists.^[9]^

TB-IGRA shows 82%–84% sensitivity and 86% specificity for intestinal TB but can yield indeterminate results, as in our patient.^[8]^ Indeterminate outcomes are more common in Asian populations, patients with low albumin, or anemia, complicating diagnosis.^[10]^ In our endemic area, TB remains a primary consideration, especially given unstandardized testing at primary care; thus, we performed AFB and PCR before starting immunosuppression. The false-negative ANA ELISA reflects its lower sensitivity (70%–80%) compared to immunofluorescence (95%–98%).^[3,11]^ Specialized ANA tests are essential when clinical suspicion remains high.

Ileocolonoscopy in LE typically reveals nonspecific, ring-shaped ulcers in the terminal ileum, consistent with our findings.^[3,4]^ In contrast, ITB usually shows transverse ulcers, a patulous ileocecal valve, post-inflammatory pseudopolyps, and scarring, often affecting fewer than 4 bowel segments.^[12]^ This ileocecal predilection in ITB relates to dense lymphoid aggregates, a neutral pH facilitating mycobacterial absorption, and local stasis.^[7]^ Biopsies in LE reveal chronic nonspecific inflammation, whereas tuberculosis may show granulomatous inflammation, although caseation is not always present.^[2,3,6]^ Endoscopic studies can help to exclude other diagnoses, but they are neither sensitive nor specific. Macroscopic examination reveals edematous, hyperemic or ischemic bowel wall appearances with or without infarction.^[3]^ Biopsies showed nonspecific chronic inflammation, consistent with LE, although vasculitis or immune complex deposition may be absent in superficial or early samples. Full-thickness biopsies, rarely performed due to perforation risk, but can reveal small-vessel vasculitis or immunoglobulin deposits, which are more specific for LE.^[3,6]^ In our case, before serologic tests were available, lymphoma involving the ileocecal valve and terminal ileum was an important differential to consider, given that this region is commonly affected and lymphoma can mimic inflammatory bowel disease when presenting with “B” symptoms. Repeated biopsies are sometimes needed to confirm lymphoma. However, serologic markers supported lupus in this case, and biopsies were negative for lymphoma, helping to rule it out. In regions where inflammatory bowel disease is prevalent, LE can also mimic Crohn disease and should be considered in the differential diagnosis when there is small intestine involvement.^[13]^

The radiologic and colonoscopic findings in this case – nonspecific ileal thickening, ulceration of the ileocecal valve, and extensive lymphadenopathy – were not diagnostic for a specific chronic disease involving the ileocecal region. In our areas, ITB is the most likely differential, but the abdominal CT images did not show the classic “target sign” of vasculitis, and the endoscopic findings were not conclusive for tuberculosis, Crohn disease, vasculitis, or lymphoma. The images in LE commonly show the “target sign” – bowel wall thickening >3 mm with separation of mucosal and muscular layers – along with submucosal edema and diffuse, non-necrotic lymphadenopathy, as seen in our patient’s perihepatic, peripancreatic, and perisplenic nodes.^[14,15]^ Conversely, ITB is characterized by necrotic lymph nodes, asymmetric wall thickening of the terminal ileum and cecum, and sometimes ascites or strictures.^[16]^ Chest CT in our patient revealed scattered interstitial changes, not typical of tuberculosis, and negative bronchoalveolar lavage and ileal biopsy PCR for *Mycobacterium tuberculosis* further supported LE. Table 1 summarizes key distinguishing features between LE and ITB.

**Table 1:** The differences between gastrintestinal tuberculosis disease and lupus enteritis.^[3,6,8,9,12,16]^

Criteria	Gastrointestinal tuberculosis	Lupus enteritis
Clinical presentation	Fever, night sweats, weight loss, abdominal pain (often right lower quadrant); history of pulmonary TB or positive PPD/IGRA.	Abdominal pain, nausea, vomiting, diarrhea; often associated with high SLE activity and other manifestations (e.g., renal, pericardial).
Imaging	Concentric, nonstratified bowel wall thickening; necrotic mesenteric lymphadenopathy; localized ileocecal involvement; ascites or strictures.	Stratified bowel wall thickening with “target sign”; comb sign (hypervascular mesentery); diffuse small bowel involvement; non-necrotic lymphadenopathy; ascites.
Endoscopy findings	Involves < 4 segments, with transverse or circumferential ulcers, patulous ileocecal valve, pseudopolyps; typical lesions often localized to the ileocecal area.	Nonspecific findings; mild mucosal edema or erythema, ulcers are rare; typically affects multiple small bowel segments without clear endoscopic hallmark.
Specific biomarker	IGRA or positive PPD has limited diagnostic value in high TB-burden countries, especially in Asia, and performs poorly in patients with hypoalbuminemia or anemia.	Low complement levels (C3 and C4), high anti-dsDNA antibody, and leucopenia.
Gold standard for diagnosis	Histopathology showing caseating granulomas; *Mycobacterium tuberculosis* detection via acid-fast staining, culture, or PCR.	Clinical diagnosis with typical CT findings, high SLE activity, and serological markers; full-thickness biopsy rarely needed for confirmation.
Therapy	Antituberculosis drugs (e.g., isoniazid, rifampicin) for 6–9 mo.	High-dose glucocorticoids and immunosuppressive agents like mycophenolate mofetil or cyclophosphamide.

CT = computed tomography, IGRA = interferon-gamma release assay, SLE = systemic lupus erythematosus.

Misdiagnosis risks are serious: immunosuppression may worsen TB, while delayed immunosuppression in LE increases complication risks. Our patient improved rapidly with methylprednisolone, and remission was maintained with azathioprine after 1 year. However, This single-case report is inherently limited in generalizability. Imaging and histologic findings were nonspecific, and the absence of full-thickness biopsy precluded definitive pathological confirmation. Further studies are warranted.

In conclusion, this case highlights the diagnostic challenges in tuberculosis-endemic regions, where LE can mimic more common conditions such as abdominal tuberculosis. Timely recognition and accurate diagnosis are crucial in these settings. Combining imaging, biopsies, and serologic tests helps to avoid inappropriate treatments and complications.

## Acknowledgments

We thank the patient and her family for their participation.

## Author contributions

**Conceptualization:** Tien Manh Huynh, Nguyen Khoi Huynh.

**Data curation:** Tien Manh Huynh, Nguyen Khoi Huynh, Tran Luong Thi Vo, Huong Tu Lam, Thinh Duc Nguyen.

**Formal analysis:** Tien Manh Huynh.

**Investigation:** Tien Manh Huynh, Nguyen Khoi Huynh.

**Methodology:** Tien Manh Huynh, Nguyen Khoi Huynh.

**Supervision:** Dong-Hoon Yang.

**Writing – original draft:** Tien Manh Huynh, Nguyen Khoi Huynh.

**Writing – review & editing:** Tien Manh Huynh, Nguyen Khoi Huynh, Dong-Hoon Yang.
